# Independent Protection and Influence of the Spike-Specific Antibody Response of SARS-CoV-2 Nucleocapsid Protein (N) in Whole-Virion Vaccines

**DOI:** 10.3390/vaccines11111681

**Published:** 2023-11-02

**Authors:** Huijie Yang, Ying Xie, Shuaiyao Lu, Yufang Sun, Kaiqin Wang, Shuyan Li, Junzhi Wang, Guoyang Liao, Changgui Li

**Affiliations:** 1 Divsion of Respiratory Virus Vaccines, National Institutes for Food and Drug Control, Beijing 102629, China; jieer6423@outlook.com (H.Y.); kqwang@nifdc.org.cn (K.W.); augustlisy@outlook.com (S.L.); 2 Institute of Medical Biology, Chinese Academy of Medical Sciences & Peking Union Medical College, Kunming 650108, China; nicole_tse@outlook.com (Y.X.); lusy@imbcams.com.cn (S.L.); liaogy@imbcams.com.cn (G.L.); 3 Graduate School, Guangzhou Medical University, Guangzhou 511495, China; 4 National Institutes for Food and Drug Control, NMPA Key Laboratory for Quality Research and Evaluation of Biological Products, Beijing 102629, China; wangjz_nifdc2014@163.com

**Keywords:** SARS-CoV-2, nucleocapsid protein, T cell response, S-specific antibody response, whole-virion vaccine

## Abstract

Of all of the components in SARS-CoV-2 inactivated vaccines, nucleocapsid protein (N) is the most abundant and highly conserved protein. However, the function of N in these vaccines, especially its influence on the targeted spike protein’s response, remains unknown. In this study, the immunization of mice with the N protein alone was shown to reduce the viral load, alleviating pulmonary pathological lesions after challenge with the SARS-CoV-2 virus. In addition, co-immunization and pre-immunization with N were found to induce higher S-specific antibody titers rather than compromise them. Remarkably, the same trend was also observed when N was administered as the booster dose after whole inactivated virus vaccination. N-specific IFN-γ-secreting T cell response was detected in all groups and exhibited a certain relationship with S-specific IgG antibody improvements. Together, these data indicate that N has an independent role in vaccine-induced protection and improves the S-specific antibody response to inactivated vaccines, revealing that an interplay mechanism may exist in the immune responses to complex virus components.

## 1. Introduction

Coronavirus disease 2019 (COVID-19) vaccines based on different technology platforms have been extensively used to reduce severe acute respiratory syndrome coronavirus 2 (SARS-CoV-2)-related morbidity and mortality [1,2,3]. Different vaccines designed using various technologies work through different mechanisms. The target protective antigen of these vaccines is typically the SARS-CoV-2 spike (S) protein, which contains the receptor-binding domain (RBD) that recognizes angiotensin-converting enzyme 2 (ACE2) to facilitate attachment to host cells. Thus, these vaccines stimulate an effective S-specific neutralizing antibody (NAb) response that blocks viral entry [4].

Compared to adenovirus vaccines and mRNA vaccines, which target the spike antigen and achieve stronger NAb responses [5,6], SARS-CoV-2 whole-virion vaccines contain multiple components, including the spike, nucleocapsid (N), membrane (M), and envelope (E) structural proteins. Of all SARS-CoV-2 virion proteins, the N protein is the most abundant and highly conserved. Although N-specific antibodies are non-neutralizing, the N protein was reported to be a representative antigen of the T cell response that can induce SARS-CoV-2-specific T cell proliferation and cytotoxicity in vaccinated rhesus macaques [7]. Moreover, one study aiming to detect the specific epitopes recognized by memory CD8^+^ T cells among patient peripheral blood mononuclear cells (PBMCs) found that these epitopes were mainly located in the SARS-CoV-2 nucleocapsid [8], and stronger T cell immunity targeting the conserved nucleocapsid over the spike protein can be elicited in individuals with infection of SARS-CoV-2 [9].

In addition, previous researchers have suggested that the N protein could be used as a target for a universal COVID-19 vaccine [3,10]. Combination of S- and N-based vaccines yields protection of both the lungs and brain, whereas a spike-based vaccine confers protection only in the lungs [11]. However, whether the natural N protein functions as an assistant or interferer in virion vaccines is unknown.

In this study, we aimed to explore the potential functions of N protein immunization alone against SARS-CoV-2 challenge in mice, mimicking the natural N protein component of SARS-CoV-2 whole-virus vaccines. In addition, due to co-exposure to the immune response in whole-virus vaccines, we focused on the N protein’s influence on the desired S-specific antibody response to elaborate the possible immune response interplay between internal components of the whole-virion inactivated vaccine and thus to extend the understanding of this classical type of vaccine.

## 2. Materials and Methods

### 2.1. Virus

The SARS-CoV-2 prototype strain (GD108#) was provided by the Institute of Medical Biology, Chinese Academy of Medical Sciences, Kunming, China.

### 2.2. Animals

Specific pathogen-free (SPF) 6~8-week-old female H11-K18-hACE2 mice (Strain NO. T037657), which were highly susceptible to SARS-CoV-2 infection, were purchased form GemPharmatech (Nanjing, China).

SPF 6~8-week-old female BALB/c mice used for immunogenicity tests were purchased from the National Institutes for Food and Drug Control (NIFDC), Beijing, China.

All animal research protocols were approved by the Institutional Animal Care and Use Committee at the National Institutes for Food and Drug Control (NIFDC), Beijing, China (No. 2022 (B) 006), and the Institute of Medical Biology, Chinese Academy of Medical Sciences, Yunnan, China (DWSP202208001). These protocols were followed in accordance with the regulations regarding the management of laboratory animals (National Science and Technology Commission no. 2 of 31 October 1988) and “guidance notes on the treatment of experimental animals” (Chinese version (2006) no. 398). All institutional guidelines for animal care and use were strictly followed throughout the study.

### 2.3. Immunization and Challenge of Mice

To assess the potential protective role of the nucleocapsid protein, SARS-CoV-2 immunization and challenge experiments were conducted at the Institute of Medical Biology, Chinese Academy of Medical Sciences National Primates Research Center, Yunnan, China. H11-K18-hACE2 mice (*n* = 11) were intraperitoneally (i.p.) immunized via a prime-boost regimen with recombinant N or S protein (Sino Biological Inc., Beijing, China, cat#40588-V08B and cat#40589-V08B1) with aluminum hydroxide (0.45 mg/mL Al(OH)_3_, Al, provided by SinoVac, Beijing, China) adjuvant dilution buffer at both week 0 and week 2. The Al adjuvant buffer was solely employed in the negative control group. Two weeks after the second vaccination, 5 mice were sacrificed to collect splenocytes, and the other 6 mice in each group were transferred to the Animal Bio-Safety Level 3 (ABSL-3) laboratory and challenged with SARS-CoV-2 virus. Mice were intranasally infected with 2 × 10^3^ TCID50 of the SARS-CoV-2 prototype strain (GD108#). All mice were euthanized at 5 days post-infection (5 dpi). Lung, turbinate, trachea, and cerebrum specimens were harvested for virus titer determination and histopathological examination.

For experiments on co-immunization, S with N or other non-relevant antigens including influenza nucleocapsid protein (Flu NP) and influenza vaccine (Flu vaccine), BALB/c mice (10 animals in each group) were vaccinated with the S protein (with Al adjuvant) alone or with S + N, S + Flu NP, and S + Flu vaccine. The information and doses of the proteins and vaccines are shown in Table 1. Two weeks after immunization, serum and splenocytes were collected.

For experiments on pre-existing immunization with N or other non-relevant antigens, including Flu NP, Flu vaccine, and varicella-zoster virus vaccine (VZV vaccine), BALB/c mice (10 animals in each group) primed with N or other non-relevant antigens at week 0, then whole-virion SARS-CoV-2 inactivated vaccine boosted at week 2. The information and doses of the proteins and vaccines are shown in Table 1. Splenocytes were collected from five mice at week 2 before being SARS-CoV-2 inactivated vaccine boosted, and serum was collected when the mice were euthanized at week 4.

To observe the immune response to N or other non-relevant antigens (Flu NP, Flu vaccine, and VZV vaccine) following immunization with the SARS-CoV-2 inactivated vaccine, BALB/c mice were immunized with 1/16 of the human dose of the whole-virion vaccine intraperitoneally (diluted with Al adjuvant) (*n* = 15) (week 0), and N or other antigens (Al adjuvant as the negative control) were administered as a booster at week 10. Serum was collected at week 10 (before boosting) and week 12 (two weeks after boosting), and mice were sacrificed at week 12 for splenocytes collection. The information and doses of the proteins and vaccines used in this study are shown in Table 1.

### 2.4. Virus Load Detection in Mouse Tissue Samples

Lung, turbinate, trachea, and cerebrum specimens of challenged mice were homogenized and lysed in 800 μL of TRIzol (Thermo, Carlsbad, CA, USA, cat#10296028). An automated nucleic acid extraction system (Kingfisher Flex, Thermo, Massachusetts, the United State) was used to extract viral RNA from a 200 μL sample, and extracted RNA was eluted in 50 μL of H_2_O (DNase/Rnase-free water, TIANGEN, Beijing, China, cat#RT121) according to the manufacturer‘s instructions. qRT-PCR was used to quantify viral RNA with the following primer/probe set for nucleocapsid: N-forward primer, 5′-GACCCCAAAATCAGCGAAAT-3′; N-reverse primer, 5′-TCTGGTTACTGCCAGTTGAATCTG-3′; and probe, 5′-FAM-ACCCCGCATTACGTTTGGTGGACC-BHQ1-3′ (designed based on the sequence recommended by the World Health Organization (WHO) and Centers for Disease Control (CDC) in China). The reaction system, procedure, and N-gene standard curve generation were chosen according to the product’s protocol (TaqMan^®^ Fast Virus 1-Step Master Mix, Thermo, Carlsbad, CA, USA, cat#4444434).

### 2.5. Histopathological Examination

Each mouse was dissected and the presence of lesions was determined at 5 dpi. Lung, trachea and cerebrum specimens were collected, fixed in 10% neutral-buffered formalin, embedded in paraffin, and treated with hematoxylin and eosin (H&E). Tissue sections were analyzed microscopically by two pathologists in a double-blind manner, and a pathology report was assembled based on a comprehensive assessment score, according to the histopathological atlas. According to the method reported in the literature [12], the mice were graded for lung inflammation, lung structural changes, and hemorrhages, and the scoring criteria for each index are shown in Table 2.

### 2.6. ELISA for Estimating Total Antigen-Specific IgG Titers

The binding antibody serum levels against the SARS-CoV-2 spike protein were assessed using ELISA. In brief, 96-well plates were coated overnight at 4 °C with 1 μg/mL recombinant S protein (Sino Biological Inc., Beijing, China). The plates were washed with 0.05% PBST and blocked with 1% BSA in PBS at 37 °C for 1 h. After that, mouse serum was diluted appropriately with 1% BSA (100 μL in each well) and then incubated in the plate at 37 °C for 1 h. The plates were washed with PBST and then incubated with horseradish peroxidase (HRP)-labeled goat anti-mouse IgG antibody (GE Health, Little Chalfont, Buckinghamshire, UK, 1:2000 dilution) for 1 h at 37 °C. After coloration, the OD values at 450 nm and 630 nm were measured with a full-wavelength microplate reader (Infinite M200PRO, TECAN, Groedig, Austria). The standard serum of SARS-CoV-2 spike IgG was collected from mice immunized with the SARS-CoV-2 inactivated vaccine and quantified via ELISA. The standard serum was applied in two replicates, each in a 2× dilution series containing 6 concentrations. Then, we used the OD and antibody titers of each standard to establish an indirect antibody titer ELISA standard curve, which was fitted using the 4-parameter logistic in SoftMax Pro software. The titer of antigen-specific IgG was calculated based on the corresponding standard curve.

### 2.7. IFN-γ ELISpot Assay

ELISpot assays were performed using a mouse Interferon-γ (IFN-γ) ELISpot kit (Mabtech AB, Nacka Strand, Sweden, cat#3321-4APW). The 96-well ELISpot plates pre-coated with IFN-γ protein were washed 4 times with sterile PBS and blocked with 200 µL/well DMEM medium containing 10% fetal bovine serum (FBS) at room temperature (RT) for 30 min. Splenocytes isolated from mice were separated and counted, then 2 × 10^5^ cells were plated into wells of prepared plates. There were 4 wells for every sample; 2 of them were stimulated with 5 µg/mL stimulus (recombinant protein N, S, NP of influenza, inactivated influenza, whole-virion, or glycoprotein E of VZV), while 2 wells without stimulations were used as negative controls. Concanavalin A (ConA) was used as a positive stimulus. Then, the plates were stimulated at 37 °C in humidified incubators with 5% CO_2_ for 24 h. The cells were removed and the plates were washed with PBS 5 times. Then, 1 µg/mL of detection antibody (diluted by PBS-0.5% FBS) was added into the wells (100 µL/well) and incubated at RT for 2 h. The plates were washed as described above, and 1 µg/mL Streptavidin-ALP (diluted by PBS-0.5%FBS) was added into the wells (100 µL/well) and the plates were incubated at RT for 1 h. The plates were washed and the ready-to-use substrate solution was filtered through a 0.45 µm filter and added to the wells (100 µL/well). They were developed until distinct spots emerged, and the reaction was stopped with water and the plates were left to dry. Spots were scanned and quantified using an AID ELISPOT reader (AID iSpot, Strassberg, Germany). The number of spots of IFN-γ T cells per 2 × 10^5^ cells were calculated by subtracting the corresponding negative control wells.

### 2.8. Statistical Analysis

Data are shown as min to max in box figure; all points represent the number of animals. Normally distributed continuous variable comparisons between two groups were analyzed using an unpaired two-tailed Student’s *t* test; for three or more groups, an adopted one-way ANOVA and a Tukey’s multiple comparisons test were used. Linear regression was used to reflect correlation of two variables by Pearson correlation coefficients, where the coefficient was indicated with the slope of the regression. Statistical significance was defined as * *p* < 0.05, ** *p* < 0.01, *** *p* < 0.001, and **** *p* < 0.0001 using GraphPad Prism 9.0.

## 3. Results

### 3.1. Nucleocapsid Immunization can Elicit the Production of IFN-γ-Secreting T Cells and Suppress the Viral Load to Alleviate Pulmonary Pathological Injury

**Figure 1:**
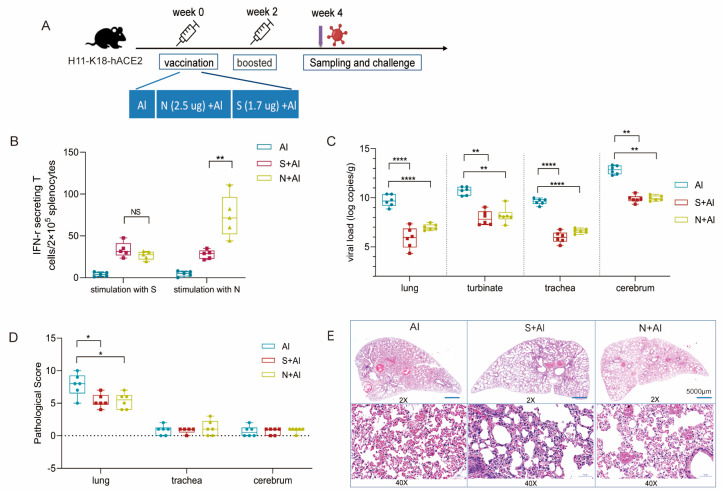
Immune response and protective effect induced by nucleocapsid (N) immunization. (**A**) Vaccination, challenge, or sample collection schedule of mice. Eleven H11-K18-hACE2 mice per group were inoculated with N (2.5 μg) + aluminum adjuvant (Al), Spike (S) protein (1.7 μg) + Al, or Al alone via intraperitoneal injection at week 0 and week 2. One group of mice (*n* = 5) were used for sampling at week 4, while in the other experiments, six mice per group were challenged for viral infection, and specimens were collected at 5 dpi. (**B**) IFN-γ-secreting T cells in the N- and S-immunized groups (*n* = 5). Splenocytes were prepared from mouse spleens two weeks after the second immunization and stimulated with recombinant N or S protein. IFN-γ-secreting T cell spots were detected via ELISpot assay with two replicates. (**C**) Viral load in mice challenged with prototype SARS-CoV-2. Lung, turbinate, trachea, and cerebrum specimens were collected at 5 dpi. SARS-CoV-2 RNA levels were measured via qRT-PCR and are expressed as viral copies. (**D**) Histopathology analysis of mouse organs on day 5 after challenge. Histograms of the pathology scores (see Table 2) of the lung, trachea, and cerebrum were produced according to the severity and extent of the histopathological lesions. (**E**) Representative images of hematoxylin and eosin (H&E)-stained lung tissues. Box plots present the distribution of data, and data were analyzed using an unpaired t test between two groups (**B**) or a one-way ANOVA for multiple groups (**C**,**D**). One spot represents one animal. * *p* < 0.05, ** *p* < 0.01, **** *p* < 0.0001, no significance (NS) *p* > 0.05.

Due to the abundance of nucleocapsid protein in SARS-CoV-2 viruses and the potential to stimulate strong T cell and humoral responses during infection, we investigated whether the N protein can be used as a sole immunogen to elicit an immune response and possible protection against SARS-CoV-2. Due to the difficulties in purifying natural N protein from whole-virion vaccines, we immunized mice with recombinant N protein and performed challenge experiments to investigate whether the N protein can independently provide protection. As presented in Figure 1A, H11-K18-hACE2 mice, which are highly susceptible to SARS-CoV-2 infection [13], were inoculated on week 0 and boosted on week 2 with recombinant nucleocapsid protein (N + Al) or recombinant spike protein (S + Al), both prepared via adsorption with aluminum hydroxide adjuvant (Al). The doses of N and S were calculated according to their amounts in the inactivated SARS-CoV-2 vaccine; they account for 50% (2.5 μg) and 35% (1.7 μg) of the human dose vaccine, respectively [14] (5μg total protein). Four weeks after vaccination, mouse splenocytes were collected and the interferon (IFN)-γ generated in response to N or S was assayed in splenocyte suspensions as a typical indicator of the T cell response. After N stimulation, splenocytes from mice vaccinated with two doses of N + Al produced significantly more specific IFN-γ than in the S + Al group and Al group (Figure 1B).

Further, to evaluate whether immunization with N alone has protective effects against SARS-CoV-2, two weeks after immunization with the second dose of Al, N + Al, or S + Al, mice were intranasally challenged with 2 × 10^3^ TCID_50_ of the prototype SARS-CoV-2 (Figure 1A). Then, lung, turbinate, trachea, and cerebrum tissues were collected from each mouse at 5 days post-infection (5 dpi), and SARS-CoV-2 genomic RNA levels, representing the replicated virus, were measured in each sample using real-time polymerase chain reactions (qRT-PCR). As shown in Figure 1C, the viral RNA copies of the S + Al and N + Al groups were 3.4~4.1 log copies/g lower than those of the Al group in all lung, turbinate, trachea, and cerebrum tissues (*p* < 0.05). N + Al immunization produced the same viral load reduction after challenge as the administration of the S vaccine did.

To analyze the histopathology, a comprehensive assessment was established according to the histopathological atlas to produce comprehensive assessment scores [12]. According to pathology examinations of the lung, the septum and alveoli were generally severely congested and hemorrhaged, and different levels of exudate were observed in the local alveolar septum (Figure 1D). Additionally, thrombosis was observed in the vascular lumen, and inflammatory cells, mainly lymphocytes and macrophages, had infiltrated the vascular lumen (Figure 1E). Compared to those in the Al group, the pathological changes in the lungs of the vaccinated groups were much less severe. However, there were no significant differences in the turbinate, trachea, and cerebrum. These results showed that immunization with N alone can induce N-specific T cell immune responses and decrease the viral load, as well as alleviate pathological lesions in mice challenged by SARS-CoV-2.

### 3.2. Nucleocapsid Protein Can Enhance the S-Specific Antibody Response

**Figure 2:**
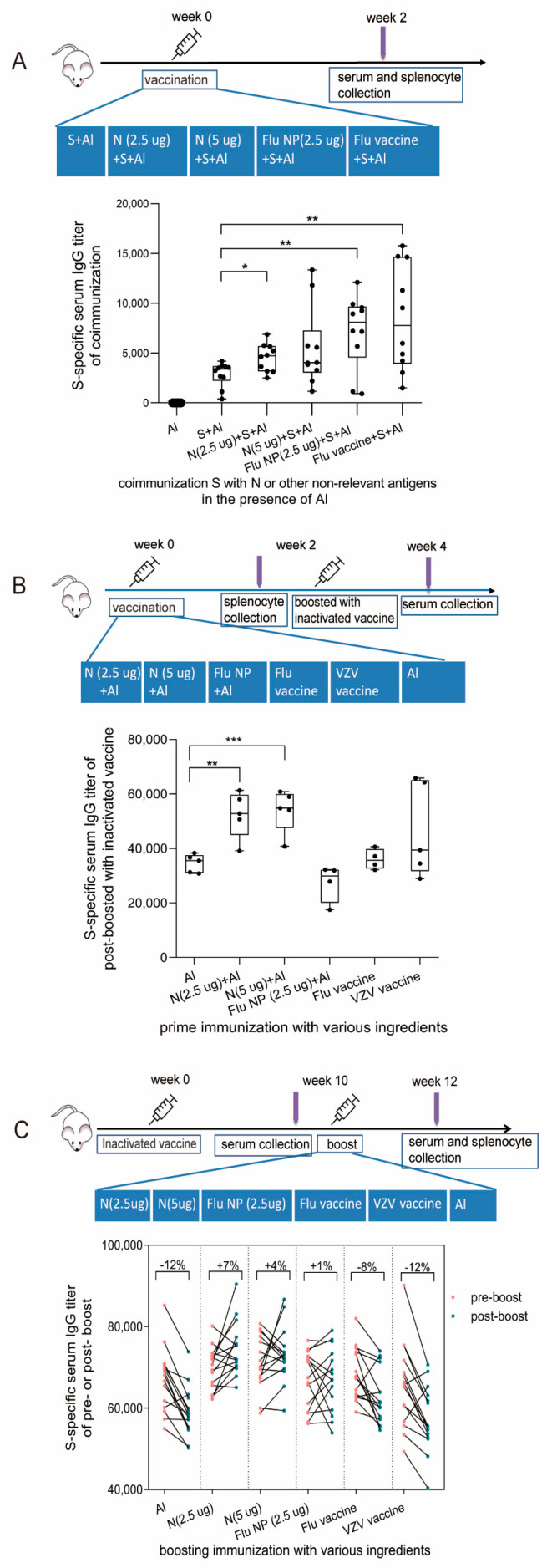
S-specific antibody responses influenced by N protein and other non-relevant antigens under different immunization schedules. (**A**) Mice were co-immunized with S and N or other non-relevant antigens. BALB/c mice were immunized with S protein alone or co-immunized with S and N protein, S and Flu nucleocapsid protein (NP), or S and Flu vaccine (contain 2.5 µg hemagglutinin protein, HA) via intraperitoneal injection on week 0 (*n* = 10). Two weeks after immunization, all mice were sacrificed; then, blood and spleens were collected (top). S-specific antibody titers of immunized mice were determined via ELISA (bottom). (**B**) Mice primed with N or other non-relevant antigens were immunized with the whole-virion SARS-CoV-2 inactivated vaccine. 10 BALB/c mice per group were primed with N + Al, Flu NP + Al, the Flu vaccine, or the varicella-zoster virus (VZV) vaccine at week 0, respectively, and all groups were boosted with a SARS-CoV-2 whole-virion inactivated vaccine at week 2. The serum was collected at week 2 before boosting (*n* = 10) and two weeks after boosting (week 4, *n* = 5). Splenocytes from 5 mice in each group were collected at week 2 before boosting, and splenocytes from another 5 mice were collected at week 4 (top). The S-specific antibody titers in the serum of mice were measured two weeks after boosting with the whole-virion inactivated vaccine (bottom). (**C**) Mice immunized with the whole-virion SARS-CoV-2 inactivated vaccine were boosted with N or other non-relevant antigens. The inactivated SARS-CoV-2 vaccine was administered at week 0 via intraperitoneal injection, and then, N and Flu NP, Flu vaccine, or VZV vaccine were used as boosters to immunize mice at week 10 (*n* = 15). Serum was collected from mice pre- and post-boosting at weeks 10 and 12. Splenocytes were isolated from mouse spleens after antigen boosting (top). S-specific IgG antibody titers in serum were measured via ELISA before and after antigen boosting (bottom), and percentages represent the change ratios of S-specific IgG antibody titers in pre- and post-boosting serum, which are calculated by the equation (post-pre)/pre titers. Data are shown as min to max in box figures; all points represent the number of animals. Normally distributed continuous variable comparisons between two groups were analyzed using a one-way ANOVA and Tukey’s multiple comparisons tests. Statistical significance was defined as * *p* < 0.05, ** *p* < 0.01, and *** *p* < 0.001 using GraphPad Prism 9.0.

To investigate the potential influence of the N protein on the S-specific antibody response, we co-immunized mice with N and S proteins. The mixture of N and S was the experimental group and S alone was the control group. Doses of 2.5 µg or 5 µg of N were used in this experiment to measure if the immune response was dose-dependent. In order to clearly observe the interaction between the two proteins, a one-dose immunization schedule was adopted in this experiment for BALB/c mice (Figure 2A). As the results show in Figure 2A (bottom), the S-specific antibody titers in the combined N and S group of mice administered with 2.5 µg N were higher than in the S alone group two weeks after immunization. Furthermore, the recombinant nucleoprotein (NP) of influenza virus and influenza vaccine (contain 2.5 µg hemagglutinin, HA), abbreviated as “Flu NP” and “Flu vaccine”, respectively, was each combined with the S protein to inoculate mice as a control. Unexpectedly, two weeks after immunization, the titers of S-specific antibodies in Flu NP + S and Flu vaccine + S groups were both higher than those of the S and S + N groups (Figure 2A), which suggests that S-specific antibody promotion is not only limited to the N protein of SARS-CoV-2.

Since co-immunization with nucleocapsid protein can enhance S-specific antibody production, we investigated the effect of pre-existing immunity induced by N protein on the immune response to whole-virion vaccine inoculation. After the primary immunization of N and other control antigens, strong IgG antibody responses of priming antigens were induced. Then, whole-virion vaccines were boosted at week 2 and the S-specific antibody levels were measured at week 4 (Figure 2B). As the results show in Figure 2B (bottom), only the group primed with N have an improved S-specific antibody response compared to other antigens, including Flu NP, the Flu vaccine, and VZV vaccine.

In addition, repetitive vaccine injection is widely applied to prevent COVID-19 [15,16]. In contrast to vaccines containing the S protein as the individual antigen, whole-virion vaccines contain all virus components, of which the N protein is the most abundant and most immunogenic. Therefore, it is also worth studying whether the S-specific antibody response is affected by repetitive exposure to the N protein. In this study, we immunized mice with the whole-virus vaccines first (week 0), and then, 10 weeks later, when the S-specific IgG antibody titers declined to a certain level, the mice were boosted with N or other non-relevant proteins and vaccines (Figure 2C). To evaluate the S immune response after boosting, we measured the S-specific antibodies in the serum before (week 10) and two weeks after (week 12) booster administration. The results show that among the 15 mice in the N (2.5 µg), N (5 µg), and Flu NP-boosted groups, 10, 8, and 9 mice exhibited an increase in S-specific antibody titers (Figure 2C, bottom), respectively. Significant S-specific antibody increases were mostly observed in groups with additional N protein exposure, especially in the 2.5 µg N group (*p* < 0.05), while a decrease was noted in the control group. Before and after boosting, the percentage of (post-pre)/pre-antibody titers was calculated (Figure 2C). The natural decay rate of S-specific antibody titers in mice in the control group was approximately −12%, whereas a titer increase of +7% was observed following being boosted with 2.5 µg N, a +4% increase was observed after being boosted with 5 µg N, and a +1% increase was observed in the Flu NP-boosted group. These results demonstrate that this rebound effect in S antibodies was mainly caused by the N protein, which is the inner protein of the SARS-CoV-2 virus. However, we did not find the dose-dependent effect for N’s boost, along with above-mentioned priming and co-administration, with similar S-specific antibody titers achieved by 2.5 µg or 5 µg N, which may be due to the “indirect boost” with heterogeneous proteins and inherent variation within animal groups.

### 3.3. T Cell Response Induced by N Shows a Certain Association with S-Specific Antibody Improvements

Since S-specific antibody responses were enhanced by co-immunization, co-immunization, pre-immunization, and N protein boosters, we explored the potential mechanism behind this. According to previous reports and our results presented in Figure 1B, N can induce a stronger T cell response than S during both infection and vaccination. In this study, we investigated the relationship between S-specific antibodies and N-specific IFN-γ-secreting T cells.

**Figure 3:**
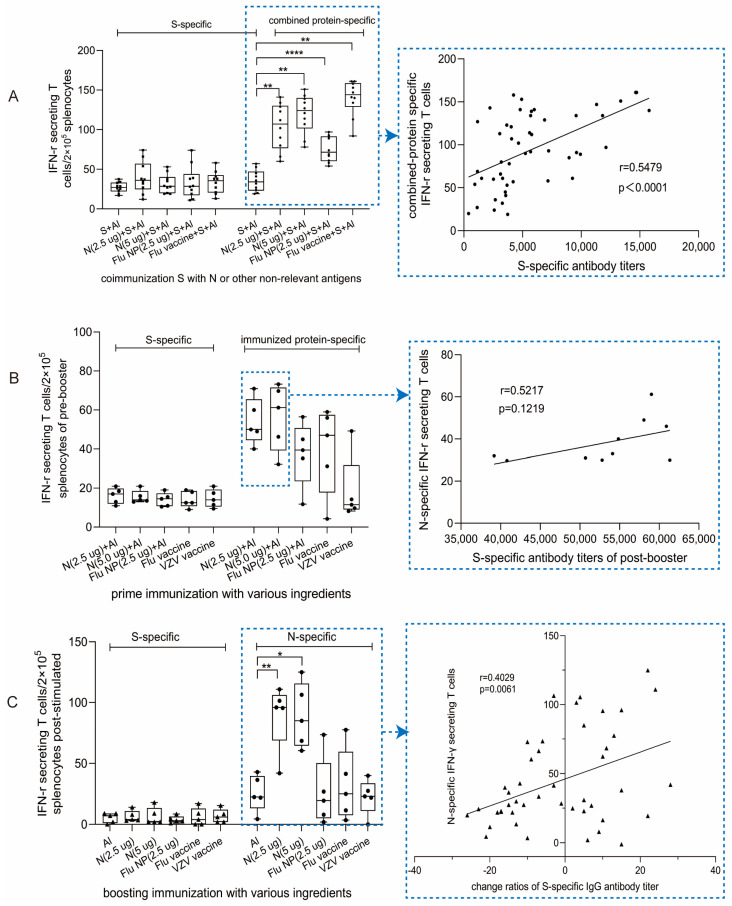
Cellular responses and their correlations with S-specific antibodies influenced by N and other non-relevant antigens under different immunization schedules. (**A**) IFN-γ-secreting T cells of co-immunized mice (as Figure 2A, top) quantified using ELISpot assays. Two weeks after co-immunization, splenocytes were isolated from mouse spleens and stimulated with S protein or the combined immunogen, including recombinant N, Flu NP, and Flu whole-virion (left). The curve showing the correlation between the S-specific antibody response (data from Figure 2A, bottom) and the corresponding specific (N, Flu NP, and Flu vaccine) T cell response for all of the mice immunized with S alone or in combination with other proteins is depicted on the right. (**B**) IFN-γ-secreting T cells among mouse splenocytes from primed mice (N, Flu NP, Flu vaccine, or VZV vaccine) (left); immune schedule details were shown in Figure 2B (top). The correlation between S-specific antibody titers after a whole-virion vaccine booster (data from Figure 2B, bottom) and the N-specific T cell response two weeks after N priming (right). (**C**) IFN-γ-secreting T cells among splenocytes from post-boost mice (left). The correlation of N-specific T cells and change ratios of S-specific antibody titers after being boosted (right). Data are shown as min to max in the box figure; all points represent the number of animals. Normally distributed continuous variable comparisons among multiple groups were analyzed using a one-way ANOVA and Tukey’s multiple comparisons tests. The r value indicates the degree of correlation following linear regression by Pearson correlation coefficients, and the slope of the regression indicates the coefficient. Statistical significance was defined as * *p* < 0.05, ** *p* < 0.01 and **** *p* < 0.0001 using GraphPad Prism 9.0.

After co-immunization with N+S, more N-specific IFN-γ-secreting T cells/2 × 10^5^ splenocytes were induced than S-specific T cells in all groups, which is in accordance with previous studies. In addition, for all combined antigens, Flu NP and Flu vaccine also induced higher specific T cells than the number of corresponding T cell spots in S-alone-immunized splenocytes (Figure 3A, left). Notably, more IFN-γ-secreting T cells were present in the Flu vaccine + S group than in the Flu NP + S group, which may be a result of the multiple antigens present in the Flu vaccine, such as HA, neuraminidase (NA), matrix protein (M), and so on. This suggests the advantages of multiple antigens in one vaccine. Together, these results indicate that not only the N protein but also the Flu NP and Flu vaccine can enhance, rather than impede, the immune response to S. Naturally, the curve between S-specific antibody titers and T cell response of combined antigens was fitted to analyze their potential correlation, and the r value obtained was 0.5479, showing that the improvement in S-specific antibody responses was partly influenced by IFN-γ secreting T cells from N and other combined antigens.

Furthermore, according to Figure 2B, S-specific antibody responses were improved by pre-immunization with N, Flu NP, the Flu vaccine, or the VZV vaccine; thus, we quantified these antigen-specific T cells to assess the influence of pre-immunization followed by whole-virion vaccination. As shown in Figure 3B, the results demonstrate that both N-specific T cells and immunogen-specific T cells were observed after primed immunization; the higher amount of N-specific T cells seems to be linked with the improvement in S-specific antibody titers, which was only demonstrated in the group primed with N (Figure 2B, below). At the same time, the r value determined by assessing the correlation curve is 0.5217, suggesting that pre-existing N-specific T cell responses improved the S-specific antibody titers to some extent after boosting with the whole-virion vaccine.

Boosting with N or other antigens after vaccination with the whole-virion vaccine leads to N-specific T cell responses (Figure 3C), corresponding with increased S-specific antibody titers (Figure 2C). After background subtraction due to primary immunization with the whole-virion vaccine, more N-specific IFN-γ-secreting T cell spots were observed in the N group than in the control group, demonstrating the N protein’s ability to induce a strong T cell immune response. In addition, to evaluate the factors causing recall of the S-antibody response and N-specific IFN-γ-secreting T cells, linear regression plots and Pearson coefficients were generated (Figure 3C, right). The r value between N-specific IFN-γ-secreting T cells and the change in the S-specific antibody titer ratio was 0.4029, indicating a weak but meaningful and important correlation between these two factors. This result suggests that the activity of T cells upon N exposure could influence the production of S-specific antibody-secreting cells, resulting in an improved antibody response.

## 4. Discussion

Although the protective immunity of Ad-N [17] or mRNA-N [18] has been reported in previous studies, our current study observed that immunization with 2.5 ug recombinant N alone, approximately calculated as one dose of the SARS-CoV-2 whole-virion vaccine, can induce an effective T cell immune response to decrease the viral load and alleviate pathological lesions in K18-hACE2 mice challenged with SARS-CoV-2. It is reasonable to suggest that the whole-virus SARS-CoV-2 vaccine can replenish cellular immunity via a strong T cell response by N proteins, improving the comprehensive effect in addition to the humoral immunity dominated by S-specific antibodies. This is in line with the report by Li et al. [19], in which the N-specific T cell response in mice was effectively induced by inactivated vaccine immunization.

Remarkably, regardless of co-immunizing with N and S alone or primed with N, both could increase S-target immune response, as even boosting with N following whole-virion vaccination was beneficial to S-specific antibody maintenance. The correlation analysis between the IFN-γ-secreting T cell responses of N- and S-specific antibody improvements suggests a weak but positive role in the target S antibody, which probably results from IFN-γ cytokines secreted by N-specific T cells.

In addition, although the Flu vaccine was originally set up as a control in this study on the influence of intrinsic and external SARS-CoV-2 virus proteins on S, its co-immunization with S induced a significantly higher S-specific antibody response than immunization with S alone. This result echoes a previous report from a phase 4 trial of a whole-virion vaccine, in which the seroconversion rate and the titer of SARS-CoV-2 neutralizing antibody in the SARS-CoV-2 + Flu vaccine group were not inferior to those in the SARS-CoV-2 vaccine group [20]. Similarly, Alsharifi et al. reported that co-administration of a whole inactivated influenza A virus vaccine and pneumococcal vaccine not only enhances pneumococcal-specific antibody responses [21] but also augments influenza A virus-specific immunity [22]. Therefore, considering the threat posed by the co-circulation of influenza and the SARS-CoV-2 virus [23], our findings support simultaneous inoculation with the two vaccines in high-transmission seasons.

As far as we know, this is the first report in which the non-target protein in the virus stimulated the targeted antigen’s immune response. Whether other kinds of typical whole-virion vaccines, e.g., Flu vaccines, have the same mechanism should be studied in the future. The exact mechanism of this kind of interplay in terms of the immune response remains unknown as there are few studies in this area. Either antigen uptake and presentation or the side effects from cytokines secreted by specific T cells could be considered for future study to understand this phenomenon. On the other hand, the improvement in the response to the S protein after administration of other antigens probably suggests weak S protein immunogenicity in whole-virion vaccine, inducing a relatively low antibody response in human populations [24]. Nevertheless, co-immunization with an inactivated virus introduces additional TLR ligands into the vaccine. These additional components, along with N within the virion, create an “adjuvant system”, rather than relying solely on alum [25,26,27].

Again, these findings confirm the existence of immune interplay between different proteins of virions in a whole-virion vaccine while extending the study to the memory aspect. After inoculation with the whole-virion vaccine, a comprehensive immune response against both spike and nucleocapsid proteins was established. The immune memory for spike or nucleocapsid proteins could be recalled after future SARS-CoV-2 infection, thus contributing to the protective function of vaccines [28]. Our results demonstrate that pre-existing specific CD8^+^ T cells from the N protein can increase S-specific antibody titers, while Flu NP, the Flu vaccine, and the VZV vaccine could not. Therefore, the ability of the pre-existing T cell immune response to increase S-specific antibody levels may be limited to the intrinsic proteins of the SARS-CoV-2 virion. Remarkably, N exposure after SARS-CoV-2 whole-virion vaccine reversed the natural decay of S-specific antibody titers. However, the immune response crosstalk between different proteins of the virus or whole-virion vaccines is complex and requires further study. For example, M and other proteins also have a dominant cellular immunogenicity that could be a potential target for SARS-CoV-2 vaccine design [29].

Notably, repeat immunization with non-target antigens may also produce certain negative effects on the immune response to target antigens [30]. Non-target antigens may cause more “antigenic original sin”, resulting in a decrease in the boosted immune response against the latest mutant strains [31], which requires more attention and balanced consideration in vaccine antigen design in the future.

Overall, our results suggest that an effective cellular immune response to N proteins induced by whole-virion vaccines may substantially prevent the development of severe SARS-CoV-2 infection. Our findings regarding the synergistic effects existing in multiple intrinsic proteins of whole-virion vaccines will fill a gap in the understanding of the complex mechanisms of these types of classical vaccines.

## Figures and Tables

**Table 1:** Proteins and vaccines used in this study.

Protein or Vaccine	Brand and Cat. Number	Dose
Recombinant SARS-CoV-2 Nucleocapsid protein (N)	Sino Biological Inc. cat# 40588-V08B	2.5 μg/5.0 μg
Recombinant SARS-CoV-2 Spike (S1 + S2) protein (S)	Sino Biological Inc. cat# 40589-V08B1	1.7 μg
Recombinant Influenza Nucleoprotein (Flu NP)	Sino Biological Inc. cat# 11675-V08B	2.5 μg
Influenza vaccine bulk	HUALAN Bio, Xinxiang, China	2.5 μg HA
VZV vaccine	Pre-clinical product, Beijing, China	Human dose
SARS-CoV-2 inactivated vaccine	SinoVac, CoronaVac	1/16 human dose

**Table 2:** Scoring scale for pathological changes in the lungs of mice.

Score	Pathological Changes
0	Clear alveolar structure, with no inflammatory infiltration
1	Mild inflammation with slight widening of alveolar septa and sparse infiltration of mononuclear cells, including monocytes and lymphocytes
2	Significant inflammation with alveolar wall thickening and increased interstitial mononuclear cell inflammatory infiltration
3–4	Significant widening of alveolar septa and increased inflammatory cell infiltration
5	Extensive exudate and septal widening, small alveolar cavity, marked septal hemorrhage, and elevated cellular infiltration in the alveolar cavity
>5	Massive cellular infiltration of the alveolar cavity, disappearance of the alveolar cavity, fusion of the intervals into a sheet, and formation of a hyaline membrane in the alveolar wall

## Data Availability

Data are available upon request from jieer6423@163.com or Nicole_Tse@outlook.com.
